# Repurposing Platinum(IV) Prodrugs to Modulate Mitochondrial Metabolism

**DOI:** 10.1021/acscentsci.3c00654

**Published:** 2023-07-12

**Authors:** Emmanouil Zacharioudakis, Raphaël Rodriguez

**Affiliations:** ^†^Department of Biochemistry, ^‡^Department of Medicine, ^§^Albert Einstein Cancer Center, ^∥^Wilf Family Cardiovascular Research Institute, and ^⊥^Institute for Aging Research, Albert Einstein College of Medicine, Bronx 10461, New York, United States; # Institut Curie, CNRS, INSERM, PSL Research University, Paris 75248, France; ∇ Equipe Labellisée Ligue Contre le Cancer, Paris 75013, France

Cisplatin is one of the most prescribed chemotherapeutic drugs used to treat solid tumors. It is a cytotoxic drug that alters DNA by forming covalent bonds with purine residues and produces toxic DNA lesions (DNA-Pt). These lesions include monoadducts and intrastrand and interstrand cross-links and induce genomic instability by acting as a physical roadblock to DNA-templated processes, such as gene transcription and DNA replication.^1^ Despite the initial success of cisplatin treatment, many patients suffer from the debilitating effects of disease progression due to intrinsic or acquired resistance to cisplatin. Previous studies have established that cancer cells activate adaptive mechanisms that confer resistance to cisplatin by alleviating genotoxic stress induced by cisplatin treatment. These mechanisms include nucleotide excision repair (NER), which can directly remove DNA-Pt and repair associated DNA damage.^1^ Alternatively, DNA-Pt can be bypassed by low-fidelity DNA polymerases during replication through a mechanism known as translesion synthesis (TLS) that enables replication of damaged DNA.^2^ Thus, there is a pressing need to identify novel platinum-based drugs and treatment regimens that can effectively overcome resistance to cisplatin. While the cytotoxic action of cisplatin is exerted through its interaction with DNA, platinum-based drugs have been shown to induce cellular stress by targeting other biomolecules (e.g., proteins). In this issue of *ACS Central Science*, Dhar and co-workers report the development of a novel cisplatin-based prodrug, namely, Platin-L, that kills prostate cancer cells by inhibiting fatty acid oxidation and subsequently decreasing mitochondrial bioenergetics.^3^

**Figure 1:**
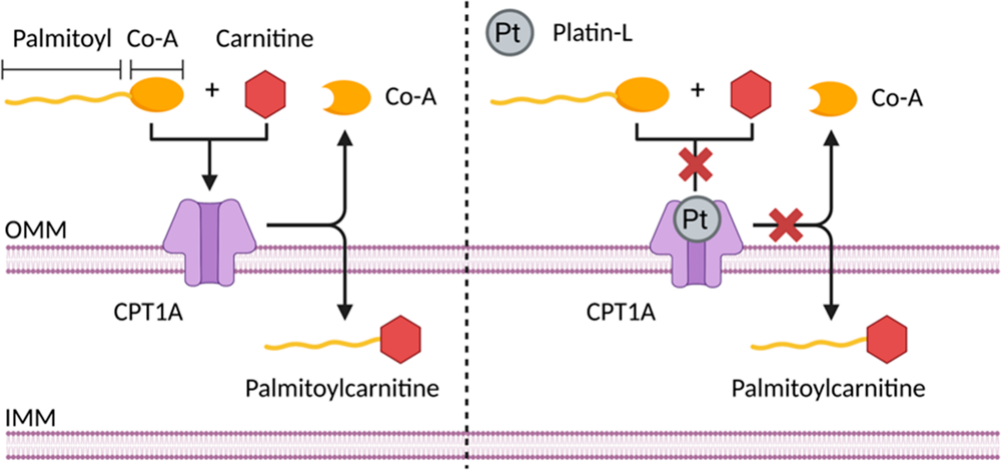
Schematic overview of the Platin-L mechanism of action. Platin-L inhibits the conversion of palmitoyl-CoA to palmitoylcarnitine by CPT1A and subsequently its uptake by mitochondria.

Platin-L is a platinum(IV) complex in contrast to the FDA-approved platinum drugs cisplatin, carboplatin, and oxaliplatin, which are platinum(II) complexes. Platinum(IV) complexes are significantly less reactive than platinum(II) complexes due to differences in their electron density and related geometry. Platinum(IV) complexes coordinate six ligands and adopt an octahedral geometry, while platinum(II) complexes coordinate four ligands and adopt a square planar geometry.^4^ To date, platinum(IV) complexes have been used as prodrugs, which are reduced in cancer cells to produce a platinum(II) complex reactive toward DNA.^4^ Interestingly, Dhar and co-workers identified that the molecular target of Platin-L is carnitine palmitoyltransferase 1A (CPT1A), an enzyme that resides on the outer mitochondrial membrane and catalyzes the transfer of an acyl group from palmitoyl-CoA to carnitine to generate palmitoylcarnitine (Figure 1).^3^ CPT1A is a component of the carnitine shuttle system that regulates the import of long-chain fatty acids (e.g., palmitate) to mitochondria where they undergo β-oxidation to generate metabolites that fuel the tricarboxylic acid (TCA) cycle and respiration.^5^ Strikingly, Platin-L accumulated in mitochondria of prostate cancer cells reduced the uptake of palmitate in mitochondria and decreased respiration. Further characterization of this mechanism revealed that prostate cancer cells rely on fatty acid oxidation for energy production and undergo apoptosis in response to Platin-L treatment. This study identified a prototype platinum(IV) prodrug that operates as a CPT1A inhibitor and induces cell death by modulating mitochondrial metabolism.

Importantly, Platin-L effectively reduced cell viability in a panel of human cancer cell lines with variable sensitivity to cisplatin. This finding suggests that targeting mitochondrial metabolism using Platin-L can be an alternative strategy to overcome cisplatin resistance that rises from the activation of DNA repair mechanisms. Strikingly, Platin-L significantly reduced tumor volume and increased survival of mice in a prostate xenograft model with acquired resistance to cisplatin treatment. Moreover, Dhar and co-workers took advantage of the favorable physicochemical properties of platinum(IV) complexes and developed a targeted drug delivery system that directed Platin-L to prostate cancer cells. To this end, Platin-L was encapsulated in biodegradable nanoparticles that were coated with an antibody that recognizes prostate specific membrane antigen (PMSA), an antigen that is highly expressed in prostate cancer cells. Using this drug delivery strategy, Dhar and co-workers were able to reduce peripheral neuropathy, a common side effect that is induced by cisplatin treatment, in mice treated with Platin-L.^3^

Overcoming resistance to cisplatin has been subject to extensive research over the past 20 years. Previous efforts led to the development of rational drug combinations that have shown promising results in preclinical models of cisplatin resistance, and some of them are under clinical evaluation. In particular, cisplatin has been combined with drugs that modulate the accessibility of genomic DNA or suppress DNA repair mechanisms.^6^ For instance, treatment of cancer cells with vorinostat, a histone deacetylase inhibitor, induced the formation of clusters of DNA-Pt by relaxing chromatin and subsequently repurposed TLS from a resistance mechanism to an apoptotic trigger.^2^ The discovery of Platin-L paves the way for the development of novel drug combinations that can overcome resistance to cisplatin by targeting metabolic dependencies in cancer cells. In line with this idea, there is a growing body of literature demonstrating that reprogramming of mitochondrial metabolism can modulate chromatin architecture and DNA damage response through retrograde signaling.^7,8^ Hence, future studies should test the therapeutic potential of Platin-L in combination with cisplatin in other types of cancer with intrinsic resistance to cisplatin that rely on fatty acid oxidation for energy production. Moreover, given that Platin-L is amenable to nanoparticle drug formulation, future drug delivery programs should focus on engineering nanoparticles to selectively target other types of tumors that rely on fatty acid oxidation. Ultimately, this study marks a step forward for the development of safer and more efficacious platinum-based therapeutics.
